# Placing the transtibial centralisation stitch at the posterior horn of the medial meniscus best restores tibiofemoral contact mechanics and extrusion following medial meniscus posterior root tears: An in vitro biomechanical study using porcine knee joints

**DOI:** 10.1002/jeo2.70217

**Published:** 2025-03-22

**Authors:** Khalis Boksh, Duncan E. T. Shepherd, Daniel M. Espino, Arijit Ghosh, Randeep Aujla, Michael E. Hantes, Tarek Boutefnouchet

**Affiliations:** ^1^ Department of Biomedical Engineering University of Birmingham Birmingham UK; ^2^ Leicester Academic Knee Unit, University Hospitals of Leicester NHS Trust Leicester UK; ^3^ Department of Orthopaedic Surgery and Musculoskeletal Trauma, University Hospitals of Larissa University of Thessaly Larissa Greece; ^4^ Department of Trauma & Orthopaedics University Hospitals of Birmingham NHS Trust UK

**Keywords:** augmentation, biomechanics, extrusion, meniscus, repair

## Abstract

**Purpose:**

To evaluate whether the position of the transtibial centralisation tunnel, on the background of an anatomical transtibial pull‐through root repair (ATPR), affects the tibiofemoral contact mechanics and meniscal extrusion for medial meniscus posterior root tears (MMPRT).

**Methods:**

Meniscal extrusion and contact mechanics were measured using two‐dimensional imaging and pressure films in 10 porcine knee joints. The posterior root was tested under six states: (1) intact; (2) MMPRT; (3) ATPR; (4) ATPR with TTC at the posterior horn (TTC‐PH); (5) ATPR with TTC midway between the PH and posterior border of medial collateral ligament (MCL) (TTC‐MID) and (6) ATPR with TTC behind the MCL (TTC‐MCL). The testing protocol loaded knees with 200‐N axial compression at four flexion angles (30°, 45°, 60° and 90°). At each angle and state, meniscal extrusion was measured as the difference in its position under load to that of the unloaded condition in the intact state. Contact area and pressure were recorded for all states at all angles and were analysed using a MATLAB programme.

**Results:**

ATPR + TTC‐PH led to greater reduction in extrusion compared to both ATPR and ATPR + TTC‐MCL at 60° and 90° (*p* < 0.02 and *p* < 0.05, respectively). ATPR + TTC‐PH improved contact area compared to ATPR at 60° (*p* = 0.037) and 90° (*p* = 0.014), and to ATPR + TTC‐MCL at 90° (*p* = 0.042). ATPR + TTC‐MID improved contact area compared to ATPR at 90° (*p* = 0.035). ATPR + TTC‐PH reduced peak contact pressure compared to ATPR at 45° (*p* = 0.046) and 60° (*p* = 0.019), and to ATPR + TTC‐MCL at 60° (*p* = 0.040). The intact meniscus, TTC‐PH and TTC‐MID repair states performed similarly across all angles with regards to contact mechanics.

**Conclusion:**

Combining ATPR with TTC‐PH provides the most appropriate biomechanical properties in reducing extrusion and improving contact mechanics following a MMPRT in porcine knees.

**Level of Evidence:**

Not applicable (laboratory study).

**Abbreviations**
ANOVA: analysis of variance
ATPR: anatomical transtibial pull‐through repair
MCL: medial collateral ligament
MMPRT: medial meniscus posterior root tear
OA: osteoarthritis
PCL: posterior cruciate ligament
PH: posterior horn
TTC: transtibial centralisation
UHMWPE: ultra high molecular weight polyethylene

## INTRODUCTION

The meniscal root is critical in maintaining meniscal function and converting axial tibiofemoral loads into hoop stresses across the meniscus [3, 25]. Biomechanical studies have shown root deficient knees to cause significant pathological extrusion [1, 5]. This results in reduced contact area on joint surfaces, increased point loading on articular cartilage, and a significant risk of osteoarthritis (OA) progression [47].

Anatomical transtibial pull‐through repair (ATPR) for medial meniscal posterior root tears (MMPRTs) are well established to counter these pathological sequelae of events, with a positive record of mid‐term to long‐term results [9, 11, 27, 50]. However, despite this, post‐operative extrusion can persist with associated progression of OA [10, 26, 44]. Recently, centralisation techniques have emerged to enhance the root repair, where the displaced meniscus is ‘centralised’ by anchoring it onto the rim of the tibial plateau prior to repair [7, 17]. This can be performed either with suture anchors [21, 22, 33], or a transtibial stitch [12, 13, 40]. A recent biomechanical study has shown promising results with the transtibial centralisation technique where the stitch is placed at the apex of the posterior horn and has advocated for its use as an adjunct for when there are concerns of persistent extrusion [12]. However, when performed arthroscopically, the apex is generally the deepest and difficult portion to repair due to its location within the knee. In posterior medial meniscus horn tears, the first repair stitch at this site is generally advocated, acting as a critical point for meniscus anchorage, thereby reducing tension across the tear, allowing better control over the meniscus position, and providing a strong foundation for further sutures [53, 65]. However, in the case of MMPRTs, 94.4% of the tears are confined within the root [29, 30]. Whilst it can therefore be assumed that positioning the transtibial stitch within the apex to be of lesser significance, recent studies suggest that following MMPRTs, the meniscus often extrudes posteromedially [6, 34, 37]. Theoretically this would be countered by positioning the centralisation stitch at the apex, even if it is at the expense of technically being more challenging.

In view of this, the purpose of this study was to quantitatively evaluate the tibiofemoral contact mechanics and extent of meniscal extrusion between various positions of the transtibial centralisation stitch in conjunction with an anatomical transtibial pull‐through root repair (ATPR). We hypothesised that its position at the apex of the posterior horn would be more effective in both (a) reducing medial meniscus extrusion and (b) restoring tibiofemoral contact mechanics to the intact state compared to the other positions.

## METHODS

### Specimen preparation

**Figure 1:**
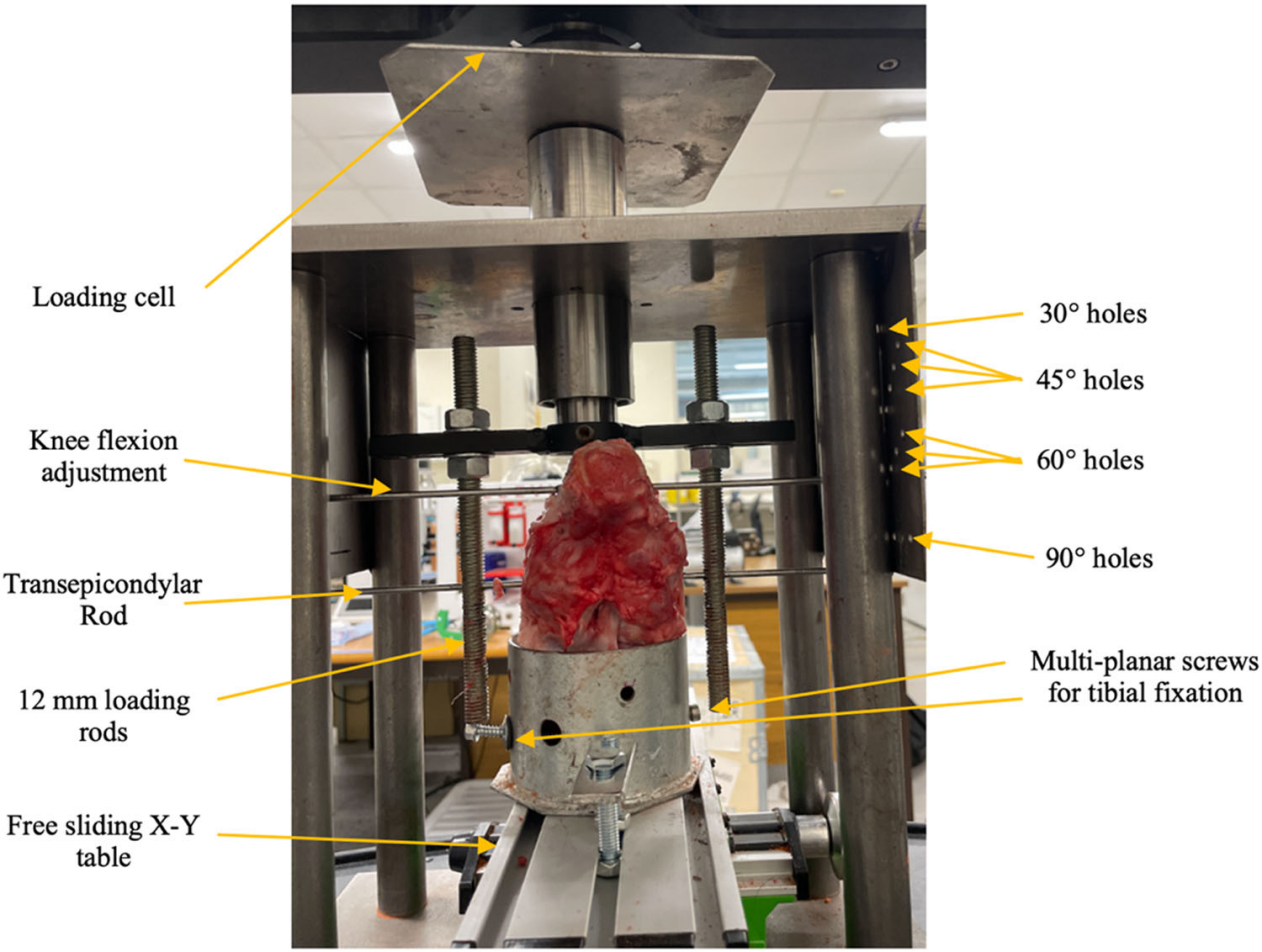
The experimental set up for biomechanical testing, with dashed lines indicating degrees of freedom.

Ten fresh‐frozen knee joints of approximately 90 kg, six‐month‐old commercially slaughtered pigs (Leicestershire, UK) with no gross evidence of meniscal damage, ligament, cartilage, or capsular tears were used in the present study. The medial compartments were used for analysis. The specimens were thawed for 24 h preceding dissection and testing. Specimens were dissected free of skin and subcutaneous tissue. All soft tissue was removed except for the knee stabilisers. However, the superficial medial collateral ligament (MCL) was cut to insert the Fuji prescale films (Fujifilm, Tokyo, Japan). Sub‐meniscal arthrotomies were performed anteromedially and posteromedially to also facilitate Fujifilm insertion, with preservation of the posterior oblique ligament and meniscotibial ligament within the deep MCL. The femur was cut 10 cm proximal and the tibia and fibula 12 cm distal from the joint line. The tibia and fibula were potted in a steel cylinder with several screws at multiplanar level fixing the tibia rigidly from multiple radial directions and at multiple superior‐inferior levels as previously described [19]. It was further augmented with Petrobond casting sand (Blackbarn Design UK), to resist smaller rotatory motions. The cylinder was then secured on an X–Y table, allowing the tibia to move freely in the anterior‐posterior, and medial‐lateral direction during loading. This free translation in the horizontal plane avoided excessive tibial constraints during axial loading to ensure physiological loading during each test (Figure 1).

### Biomechanical set up

The prepared specimen was fixed to a customised jig and secured to the BOSE ELF 3300 dynamic testing machine (Waters, Milford, MA, USA) (Figure 1). This was on the femoral side whereby a 6.5‐mm transepicondylar tunnel, parallel to the tibial plateau, was drilled through the medial and lateral femoral condyles, and a 6.5‐mm transfix pin was passed within this tunnel and secured to the jig. The load was applied through two threaded rods 12 mm in diameter, one on the medial and one on the lateral side of the knee, transmitting the axial load to the transepicondylar rod (Figure 1). The threaded rods could be adjusted in the anteroposterior and superior‐inferior directions to apply balanced load to the medial and lateral sides of the transepicondylar rod and to the femur [19]. A 4.5‐mm proximal parallel tunnel was reamed through the femoral shaft with a 4.5 mm guide pin inserted through here and the custom jig to allow for selection of flexion angles during testing (Figure 1). This set‐up was in keeping with previous studies [15, 31, 51, 52]. Previous biomechanical studies have shown that load‐bearing force during knee extension is concentrated on the anterior medial meniscus and gradually shifts to the posterior medial meniscus on deeper flexion [63, 64]. In view of this 30°, 45°, 60° and 90° angles were selected. An axial compressive force of 200 N was selected based on previous studies showing this to be large enough to yield clinically significant findings in porcine knees [2, 23, 28, 49].

### Experimental conditions

Each knee was subjected to ten testing conditions for the medial meniscus: (1) intact (no surgery); (2) type 2A MMPRT [30] (3) ATPR; (4) ATPR + transtibial centralisation at apex of posterior horn (TTC‐PH); (5) ATPR + transtibial centralisation at the posterior border of the medial collateral ligament (TTC‐PMCL) and (6) ATPR + transtibial centralisation midway between the PH and PMCL (TTC‐MID). Previous studies have shown a MMPRT generally extrudes posteromedially if it remains undetected or undergoes delayed repair [34, 37]. Furthermore, Daney et al reported the meniscus to be more susceptible to extrusion at the posterior border of the MCL [12]. These reports determined the conditions 5 and 6. Randomisation of the sequence order was performed between conditions 3, 4, 5 and 6.

Based on a previous study [2], a prior power analysis (power 0.8, *α* = 0.05) and an estimated detectable difference of 2.0 for meniscal extrusion determined that a sample size of at least five subjects in each group was required for valid comparison. The sample size within this study included 10 subjects each in the six states.

The MMPRT was created using a Number 11 scalpel blade (Swann‐Morton, Sheffield, England) immediately at the bony root attachment site, which transected the main and the shiny white fibre attachments.

**Figure 2:**
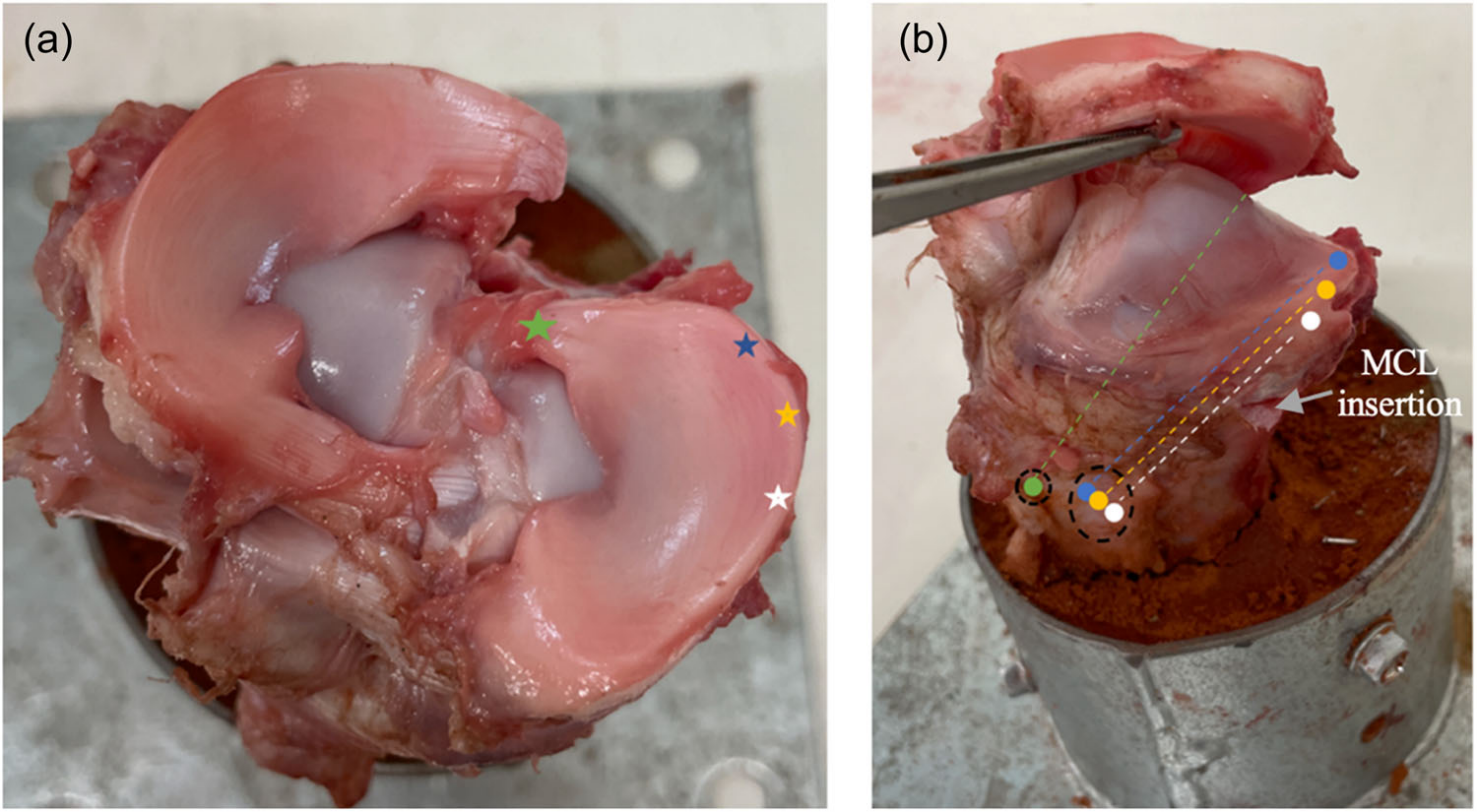
Positions of the root repair, centralisation repairs, their respective tunnels and fixation on the tibial side in a porcine knee (dissected for educational purposes, not used in final testing). (a) green star: location site for the ATPR and respective tape repair; blue star: location site for TTC‐PH; yellow star: location site for TTC‐MID; white star: location site for TTC‐PMCL (b) green linear dash: orientation of ATPR drilling with exit at anteromedial cortex (respective coloured circle), tied over button (black circular dash). *Note*: Entry point at tibial attachment site not shown. Blue (TTC‐PH), yellow (TTC‐MID) and white (TTC‐PMCL) linear dash: orientation of TTC drilling with exit at more medial anteromedial cortex (respective coloured circles), tied over separate cortical button (black circular dash). Grey arrow, MCL insertion. ATPR, anatomical transtibial pull‐through repair; MCL, medial collateral ligament; MID, midway; PH, posterior horn; PMCL, posterior border of the medial collateral ligament; TTC, transtibial centralisation.

For the ATPR condition, a single 3.2 mm transtibial tunnel was placed at the posterior root attachment site (Figure 2a). Using a simple stitch technique, two UHMWPE tapes (2 mm UltraTape; Smith and Nephew) was passed through the meniscal root 5 mm medial to its transection. The sutures were placed into a looped nitinol wire and passed down the tunnel and firmly tensioned over a Tightrope button (Arthrex) using a surgeon's knot followed by 5 half hitches on alternating posts of the fixation button (Figure 2b). These surgical steps are as described in a previous study [32], but Tapes rather than sutures and a single rather than two tunnel root repair was used in our study.

The TTC‐PH centralisation was performed in line with previous studies, but again we used Tape rather than sutures [12, 13]. Two 2 mm UltraTape (Smith and Nephew) was passed through the peripheral meniscus at the mid‐point between the posterior root attachment and the posterior border of the MCL (Figure 2a). The tape was passed through the meniscus from the tibial to femoral side and then through the femoral to tibial side to create a double‐loaded construct. This was shuttled through a 3.2‐mm centralisation tunnel, which was created from 3 mm inside the articular border (Figure 2b) and exiting out of the anteromedial cortex 2 cm medial to the ATPR tunnel. This was in keeping with a previous study [12]. Like the ATPR technique, the suture was tied but over a second fixation button using the same knotting technique described.

The TTC‐MID centralisation stitch and its respective tunnel was in between the posterior border of the MCL and the apex of the posterior horn (Figure 2a). The TTC‐MCL stitch and its respective tunnel was performed immediately behind the MCL (Figure 2a). Except for the orientation of the centralisation tunnels, the number of tapes, fixation configuration, tunnel size, exit on the anteromedial cortex and tibial fixation construct was as described for that of the TTC‐PH centralisation technique.

### Measurement of medial meniscal extrusion (MME)

**Figure 3:**
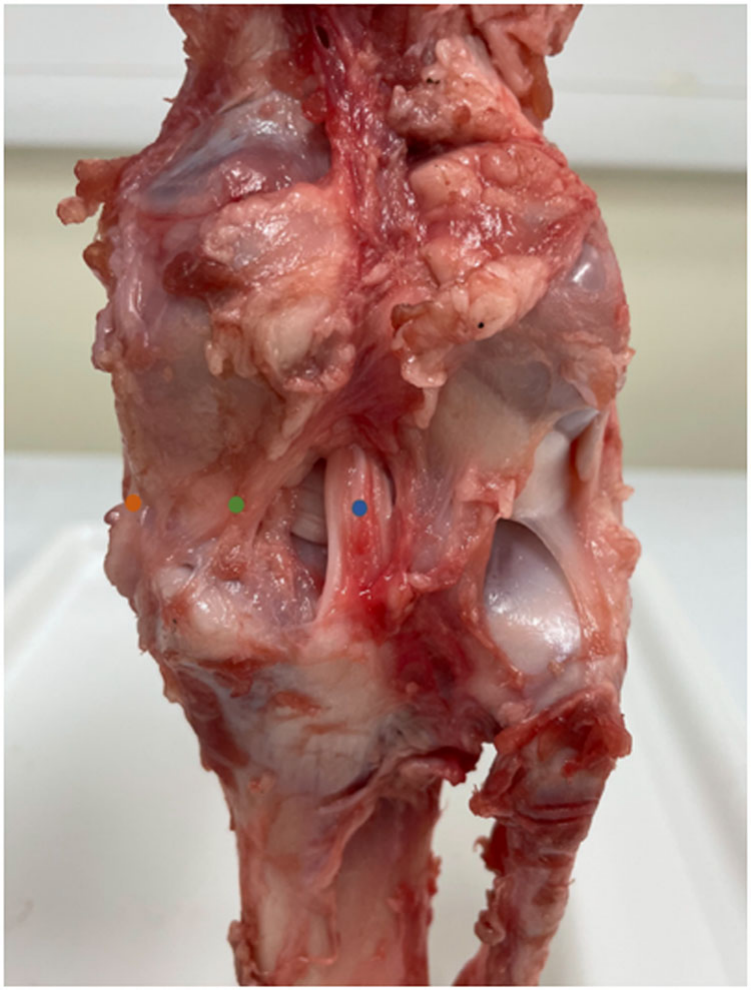
Position of the 2 mm markers at the medial meniscus in the posterior view of a left porcine knee Blue circle, first marker at the posterior border of posterior cruciate ligament (PCL); green circle, second marker at the posteromedial edge; orange circle, third marker at the posterior border of MCL.

Two‐dimensional digital photography and ImageJ (version 1.54, Rasband, W.S, USA, National Institutes of Health, Bethesda, Maryland, USA) software was used to measure medial meniscus extrusion. This software has high test‐reliability [60], with no difference in precision with that of digital calipers, often considered the ‘gold standard’ measuring technique [38]. Three sharp 2 mm markers were pinned to the medial meniscus at 3 points: the posterior marker at the centre of the tibial attachment of the posterior cruciate ligament (PCL) (first marker), the medial marker on the posterior border of the MCL (third marker), and one marker in between the two at the posteromedial edge (second marker) (Figure 3). A 2 mm scale bar was added within the field of view which was used to calibrate the images to convert the distance between markers from pixels into mm on ImageJ. Meniscal extrusion was measured for the second and third marker as the difference between the marker position with application of a 200 N compression loading and position in the unloaded condition in the intact knee. This is in keeping with a previous study [2].

### Contact area and force measurements

Tibiofemoral contact mechanics were measured with the use of Fujifilm (Tokyo, Japan). Following the protocols previously described, the packets were cut to size and sealed to prevent the incursion of moisture up to three to seven days prior to the test date [35, 45].

Prior to load application, the Fujifilm was inserted between the anteromedial and posteromedial arthrotomies as described, and positioned beneath the meniscus, covering the tibial plateau. Load was applied for two minutes to comply with the manufacturer's recommendations to allow accurate quantification of the pressures. For each loading condition, one packet of low‐pressure range and one packet of super low pressure range Fujifilm were used. A total of ten trials were performed using Fuji film for all testing states at each flexion angle.

After loading, the Fujifilm was scanned into a customised MATLAB programme (Mathworks, Natwick, Massachusetts) based on the optical density of the scanned Fujifilm and a fifth‐order polynomial developed from Fuji film calibration data.

### Statistical analysis

A one‐way repeated measures analysis of variance was applied to analyse the differences in mean values between the dependent variables (medial meniscus extrusion, peak contact pressure and area) recorded during tests across all flexion angles among the six meniscal conditions (independent variable). The intact knee was selected as the reference condition to identify statistically significant differences with meniscus extrusion with the other testing conditions. Normality and homogeneity assumptions were met and in view of this, Tukey post hoc comparisons between all pairwise combinations of knee states were performed.

We considered a value of less than 0.05 to be statistically significant. All data are presented as means with 95% confidence interval. The statistic software IBM SPSS V.29 (International Business Machines Corporation, Armonk. NY, IBM Corp, 2024) was used for all plots and analyses.

## RESULTS

### Medial meniscal extrusion

**Figure 4:**
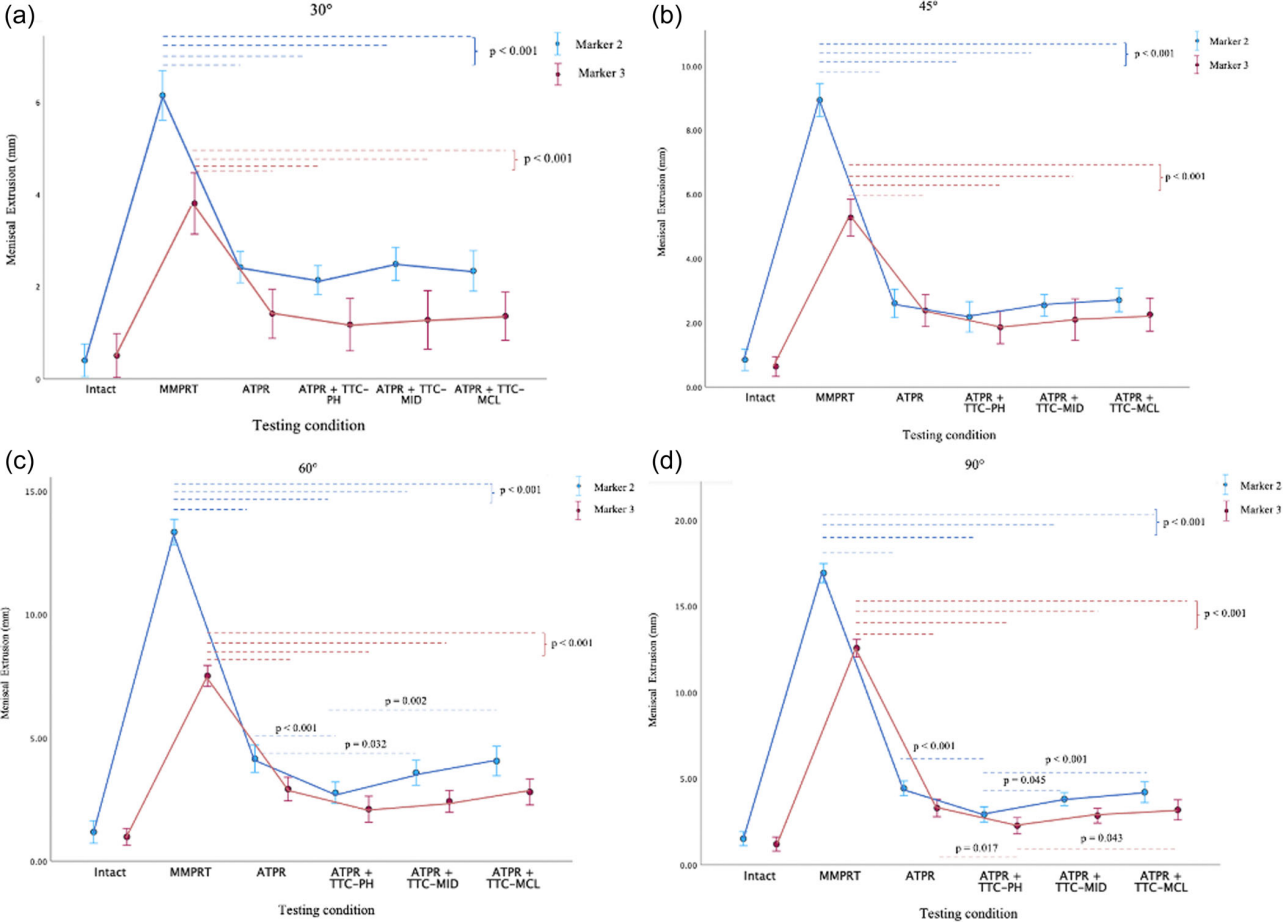
The extent of medial meniscus extrusion at two markers of the medial meniscus for each testing state at (a) 30°; (b) 45°; (c) 60°; and (d) 90°. The mean values with 95% confidence intervals are shown. All labelled significant differences are *p* < 0.05.

At all flexion angles, MMPRT led to significant meniscal extrusion from the intact state, at both markers two and three (*p* < 0.001). All fixation methods significantly reduced extrusion from the torn state, with no difference between the techniques at 30° and 45° (Figure 4a,b). At 60°, ATPR with TTC–PH led to greater reduction in extrusion in comparison to isolated ATPR (*p* < 0.001) and ATPR with TTC‐MCL (*p* = 0.002) at Marker 2 (Figure 4c). Similar results were also observed at 90°, with significant reduction in extrusion also observed at Marker 3 (Figure 4d). Furthermore, ATPR + TTC‐MID performed better than ATPR at 60° (*p* = 0.032) (Figure 4c). Other than at Marker 2 at 90° (Figure 4d), ATPR + TTC‐MID led to similar reduction in meniscal extrusion to that of ATPR + TTC‐PH at both markers through all flexion angles. No significant differences were observed in reducing meniscal extrusion when comparing isolated ATPR to that in combination with TTC‐MCL. Quantitative data is synthesised in Table 1.

**Table 1:** Qualitative data for the extent of meniscal extrusion at two markers of the medial meniscus under each testing state.

	Knee position	Meniscal extrusion for each knee condition (mm)						*p* value
		Intact	MMPRT	ATPR	ATPR + TTC‐PH	ATPR + TTC‐MID	ATPR + TTC‐MCL	
Marker 2	30°	0.40 ± 0.50^a^	6.15 ± 0.75	2.42 ± 0.47^a^	2.14 ± 0.44^a^	2.49 ± 0.50^a^	2.34 ± 0.61^a^	<0.001
		(0.12–0.82)	(5.61–6.68)	(2.08–2.76)	(1.83–2.45)	(2.13–2.85)	(1.90–2.78)	
	45°	0.85 ± 0.47^a^	8.93 ± 0.71	2.61 ± 0.63^a^	2.19 ± 0.66^a^	2.55 ± 0.47^a^	2.71 ± 0.51^a^	<0.001
		(0.52–1.19)	(8.42–9.44)	(2.17–3.05)	(1.71–2.66)	(2.21–2.88)	(2.34–3.07)	
	60°	1.18 ± 0.64^a^	13.33 ± 0.72	4.15 ± 0.77^a^	2.77 ± 0.61^a^^,^ b ^,^ c	3.12 ± 0.80^a^ ^b^	4.06 ± 0.83^a^	<0.001
		(0.73–1.64)	(12.82–13.85)	(3.60–4.70)	(2.34–3.20)	(2.54–3.70)	(3.46–4.65)	
	90°	1.51 ± 0.57^a^	16.91 ± 0.77	4.43 ± 0.60^a^	2.92 ± 0.62^a^^,^ b ^,^ c ^,^ d	3.81 ± 0.53^a^	4.21 ± 0.84^a^	<0.001
		(1.11–1.92)	(16.36–17.47)	(4.00–4.86)	(2.48–3.36)	(3.43–4.19)	(3.61–4.81)	
Marker 3	30°	0.50 ± 0.66^a^	3.80 ± 0.93	1.41 ± 0.74^a^	1.17 ± 0.80^a^	1.28 ± 0.89^a^	1.36 ± 0.73^a^	<0.001
		(0.27–0.98)	(3.14–4.47)	(0.88–1.94)	(0.60–1.74)	(0.64–1.91)	(0.83–1.88)	
	45°	0.64 ± 0.43^a^	5.28 ± 0.80	2.38 ± 0.69^a^	1.86 ± 0.71^a^	2.10 ± 0.90^a^	2.26 ± 0.72^a^	<0.001
		(0.33–0.94)	(4.70–5.85)	(1.89–2.88)	(1.35–2.37)	(1.45–2.74)	(1.74–2.77)	
	60°	0.98 ± 0.47^a^	7.50 ± 0.60	2.92 ± 0.67^a^	2.11 ± 0.74^a^	2.42 ± 0.62^a^	2.80 ± 0.73^a^	<0.001
		(0.65–1.32)	(7.08–7.93)	(2.44–3.40)	(1.58–2.63)	(1.98–2.86)	(2.28–3.33)	
	90°	1.20 ± 0.57^a^	12.56 ± 0.72	3.29 ± 0.70^a^	2.26 ± 0.66^a^ ^b^ ^c^	2.85 ± 0.60^a^	3.18 ± 0.82^a^	<0.001
		(0.79–1.60)	(12.05–13.07)	(2.79–3.79)	(1.80–2.73)	(2.42–3.27)	(2.60–3.77)	

*Note*: Marker 2 was placed at the posterior border of the MCL. Marker 3 was placed 10 mm behind Marker 2, at the posteromedial edge of the MCL. The mean values with standard deviation, and 95% confidence intervals are shown.

Abbreviations: ATPR, anatomical transtibial pull‐through repair; MCL, medial collateral ligament; MID, midway; PH, posterior horn; TTC, transtibial centralisation.

^a^ Statistically significant, comparison to torn state (*p* < 0.001).

^b^ Statistically significant, comparison to ATPR (TTC‐PH: *p* = 0.001, *p* < 0.001 and *p* = 0.017 at Marker 2 60°, Marker 2 90° and Marker 3 90°, respectively. TTC‐MID: *p* = 0.032 at Marker 2 60°).

^c^ Statistically significant, comparison to ATPR + TTC‐MCL (TTC‐PH: *p* = 0.002, *p* < 0.001 and *p* = 0.043 at Marker 2 60°, Marker 2 90° and Marker 3 90°, respectively.

^d^ Statistically significant, comparison to ATPR + TTC‐MID (TTC‐PH: *p* = 0.045 at Marker 2 90°).

### Mean contact area of the medial compartment

**Figure 5:**
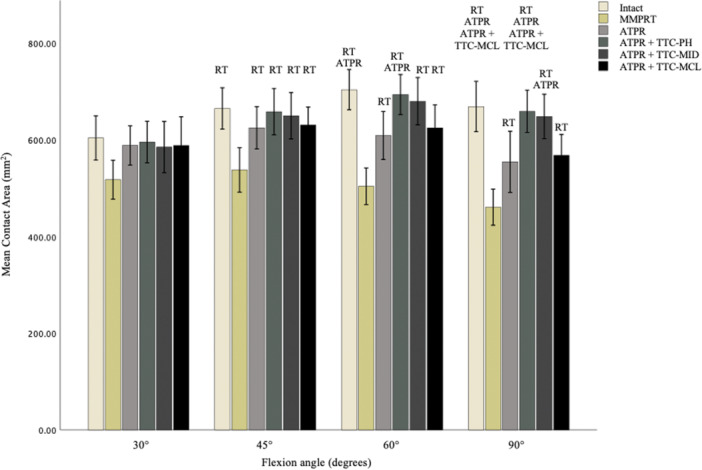
Medial compartment contact area with native meniscus, meniscal root tear and tunnel conditions. ATPR, significant from anatomical transtibial pull through repair; ATPR + TTC‐MCL, significant from root repair with centralisation tunnel behind the medial collateral ligament; RT, significant from root tear condition.

Except for 30°, the MMPRT significantly reduced contact area compared to the native state and all repair states across the remaining flexion angles (Figure 5). Isolated ATPR was unable to restore contact area to the intact state at 60° (*p* = 0.016) and 90° (*p* = 0.005). This was also the case for ATPR + TTC‐MCL at 90° (*p* = 0.018). ATPR with either TTC‐PH or TTC‐MID restored contact area to the intact state at all flexion angles, with no significant differences between the two. However, ATPR + TTC‐PH significantly improved contact area compared to isolated ATPR at 60° (*p* = 0.037) and 90° (*p* = 0.014), and to ATPR + TTC‐MCL at 90° (*p* = 0.042). In contrast, ATPR + TTC‐MID significantly improved contact area only to that of isolated ATPR at 90° (*p* = 0.035). No significant differences were observed in improving contact area when comparing isolated ATPR to that in combination with TTC‐MCL. Quantitative data is synthesised in Table 2.

**Table 2:** Mean contact area in the medial compartment at various flexion angles under each testing condition.

Knee position	Mean contact area for knee condition (mm^2^)						
	Intact	MMPRT	ATPR	ATPR + TTC‐PH	ATPR + TTC‐MID	ATPR + TTC‐MCL	*p* value
30°	604.3 ± 64.2	517.5 ± 56.6	588.8 ± 57.2	595.9 ± 60.4	585.4 ± 74.4	588.5 ± 84.0	*p* = 0.07
	(558.4–650.2)	(477.1 ‐ 558.0)	(547.9–629.7)	(552.7–639.0)	(532.2–638.7)	(528.5–648.6)	
45°	665.6 ± 59.7^a^	537.8 ± 64.7	625.5 ± 61.4^a^	658.9 ± 67.0^a^	650.6 ± 67.2^a^	631.4 ± 52.0^a^	<0.001
	(622.9–708.2)	(491.6 ‐ 584.1)	(581.6–669.4)	(610.9–706.8)	(602.5–698.7)	(594.2–668.6)	
60°	704.3 ± 57.9^a^^,^ b	503.9 ± 53.1	609.4 ± 69.8^a^	694.4 ± 57.5^a^^,^ b	680.5 ± 68.2^a^	625.4 ± 66.8^a^	<0.001
	(662.9–745.7)	(465.9–541.9)	(559.5–659.4)	(653.3–735.5)	(631.8 ‐ 729.3)	(577.7–637.2)	
90°	669.5 ± 72.72^a^^,^ b ^,^ c	460.5 ± 52.0	554.7 ± 88.9^a^	659.6 ± 61.3^a^^,^ b ^,^ c	649.1 ± 64.3^a^^,^ b	568.0 ± 61.2^a^	<0.001
	(617.5–721.6)	(423.3–497.7)	(491.1–618.3)	(615.8–703.4)	(603.0–695.1)	(524.2–611.8)	

*Note*: The mean values with standard deviation and 95% confidence intervals are shown.

Abbreviations: ATPR, anatomical transtibial pull‐through repair; MCL, medial collateral ligament; MID, midway; PH, posterior horn; TTC, transtibial centralisation.

^a^ Statistically significant, comparison to torn state (*p* < 0.05).

^b^ Statistically significant, comparison to ATPR (Intact: *p* = 0.016 and *p* = 0.005 at 60° and 90°. ATPR + TTC‐PH: *p* = 0.037 and *p* = 0.014 at 60° and 90°. ATPR + TTC‐MID: *p* = 0.035 at 90°).

^c^ Statistically significant, comparison to TTC‐MCL (Intact: *p* = 0.018 at 90°. ATPR + TTC‐PH: *p* = 0.042 at 90°).

### Peak contact pressure of the medial compartment

**Figure 6:**
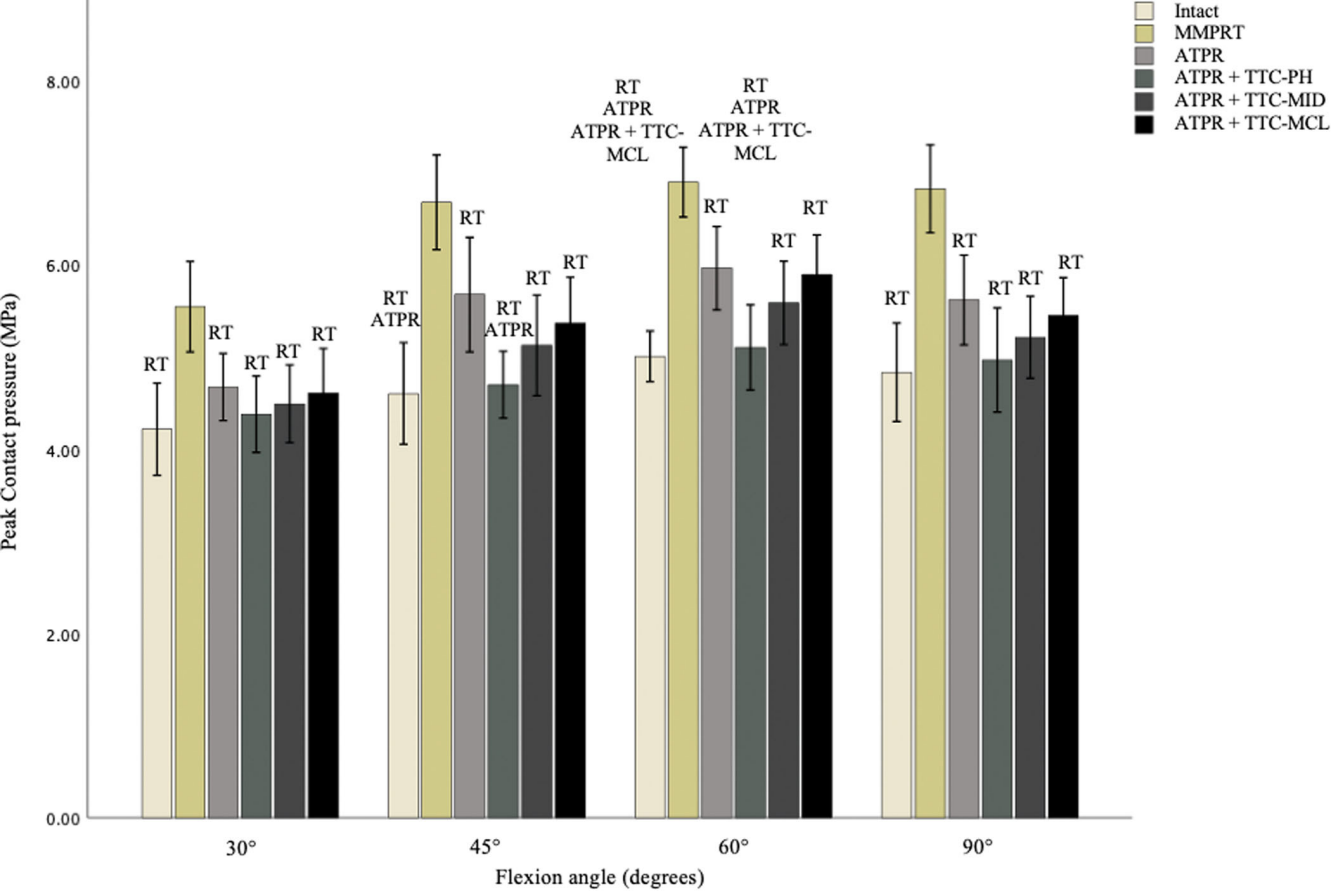
Medial compartment peak contact pressure with native meniscus, meniscal root tear and tunnel conditions. ATPR, significant from anatomical transtibial pull through repair; ATPR + TTC‐MCL, significant from root repair with centralisation tunnel behind the medial collateral ligament; RT, significant from root tear condition.

At all flexion angles, MMPRT significantly increased peak contact pressure compared to the native state and all repair conditions (Figure 6). Isolated ATPR was unable to restore peak contact pressure to the intact state at 45° (*p* = 0.021) and 60° (*p* = 0.007). This was also the case for ATPR + TTC‐MCL at 60° (*p* = 0.015). ATPR with either TTC‐PH or TTC‐MID restored peak pressure to the intact state at all flexion angles, with no significant differences between the two. However, ATPR + TTC‐PH significantly reduced peak pressure compared to isolated ATPR at 45° (*p* = 0.046) and 60° (*p* = 0.019), and to ATPR + TTC‐MCL at 60° (*p* = 0.04). In contact, ATPR + TTC‐MID had similar peak pressure levels to that of isolated ATPR, and when combined with TTC‐MCL across all flexion angles. Furthermore, no significant differences were observed in peak pressure levels between isolated ATPR and when combined with TTC‐MCL. Quantitative data is synthesised in Table 3.

**Table 3:** Mean peak contact pressure in the medial compartment at various flexion angles under each testing condition.

Knee position	Peak contact pressure (MPa) for knee condition						
	Intact	MMPRT	ATPR	ATPR + TTC‐PH	ATPR + TTC‐MID	ATPR + TTC‐MCL	*p*‐Value
30°	4.22 ± 0.70^a^	5.55 ± 0.69	4.68 ± 0.51^a^	4.38 ± 0.58^a^	4.50 ± 0.59^a^	4.61 ± 0.67^a^	<0.001
	(3.72–4.72)	(5.06–6.04)	(4.31–5.04)	(3.97–4.80)	(4.07–4.92)	(4.13–5.10)	
45°	4.61 ± 0.77^a^^,^ b	6.69 ± 0.72	5.68 ± 0.87^a^	4.70 ± 0.51^a^^,^ b	5.13 ± 0.76^a^	5.37 ± 0.70^a^	
	(4.06–5.16)	(6.16–7.20)	(5.06–6.30)	(4.34–5.06)	(4.59–5.67)	(4.87–5.87)	<0.001
60°	5.01 ± 0.51^a^^,^ b ^,^ c	6.91 ± 0.53	5.97 ± 0.64^a^	5.11 ± 0.63^a^^,^ b ^,^ c	5.59 ± 0.63^a^	5.90 ± 0.61^a^	<0.001
	(4.74–5.49)	(6.53–7.41)	(5.51–6.42)	(4.65–5.57)	(5.14–6.04)	(5.46–6.33)	
90°	4.84 ± 075^a^	6.83 ± 0.67	5.62 ± 0.68^a^	4.97 ± 0.79^a^	5.21 ± 0.62^a^	5.45 ± 0.57^a^	<0.001
	(4.30–5.37)	(6.35–7.31)	(5.13–6.11)	(4.41–5.53)	(4.78–5.66)	(5.04–5.86)	

*Note*: The mean values with standard deviation, and 95% confidence intervals are shown.

Abbreviations: ATPR, anatomical transtibial pull‐through repair; MCL, medial collateral ligament; MID, midway; MMPRT, medial meniscus posterior root tear; PH, posterior horn; TTC, transtibial centralisation.

^a^ Statistically significant from MMPRT (*p* < 0.05).

^b^ Statistically significant from ATPR (Intact: *p* = 0.021 and *p* = 0.007 at 45° and 60°. ATPR + TTC‐PH: *p* = 0.046 and *p* = 0.019 at 45° and 60°).

^c^ Statistically significant, comparison to TTC‐MCL (Intact: *p* = 0.015 at 60°. ATPR + TTC‐PH: *p* = 0.04 at 60°).

## DISCUSSION

The most important finding of this study was that positioning the transtibial centralisation stitch at the apex of the posterior horn (TTC‐PH) provided the best combination of biomechanical properties, in reducing meniscal extrusion and peak contact pressure, whilst also improving contact area across the tibiofemoral joint following a medial meniscus posterior root tear. Therefore, the TTC‐PH should be considered as an adjunct procedure where extrusion persists following root tear repair with the conventional ATPR technique.

As the flexion angle increases, the meniscus bears more weight posteriorly [66, 68]. Any resultant movement will cause posteromedial extrusion, which is thought to be the physiological direction owing to the bony anatomy of the tibial plateau, neighbouring PCL, and geometry of the medial tibial plateau and femoral condyles [6, 18, 34, 37]. Positioning the centralisation stitch at the posterior horn can counter this direction of motion, whilst maintaining the circular traction along the circumferential collagen fibrils within the meniscus [14]. This is important in keeping the meniscus tension constant, enabling it to sufficiently absorb hoop stresses and prevent radial deformation [58, 59]. In contrast, these load distribution properties are hindered when the centralisation stitch is posterior to the MCL, as its location cannot effectively counter parts of the meniscus scarring into the posterior capsule. This can subsequently cause increased cartilage deformation, local stress and point loading [51, 59]. Therefore, this may potentially underline why this centralisation technique led to greater extrusion, particularly towards pathological levels ( > 3 mm) [61], and higher contact pressure and lower contact area compared to the posterior horn centralisation at the mid to higher flexion angles.

Whilst our results showed the addition of a centralisation stitch at the apex of the posterior horn to lead to greater reduction of extrusion at 60° and 90°, greater contact area at 60° and 90°, and greater reduction in peak pressure at 45° and 60° compared to an isolated ATPR, this was in stark contrast to that reported by Daney et al. [12] who showed no differences between the two techniques across all flexion angles. It is likely the use of two UHMWPE tapes used in the present study, with their higher load to failure and stiffness compared to the suture counterpart used by Daney et al. to have enhanced the biomechanical properties of the centralisation technique [4, 41, 46, 55]. Furthermore, the wider surface area of the tape will have reduced the ‘bungee effect’ secondary to the vertical motion of the construct within the root repair and centralisation tunnel [8], thereby providing further peripheral stabilisation of the extruded meniscus and improvement in tibiofemoral mechanics.

Arthroscopically placing a centralisation stitch in the posterior horn is not without its technical challenges. Visualisation and manoeuvrability of instruments may be limited by the tight spaces deep within the joint for posterior horn access, leading to the requirement of specialised devices for effective navigation within this confined space [54]. Furthermore, adaptations such as transseptal portals or modified techniques to facilitate access may be necessary, whilst simultaneously taking care to not damage the PCL and neurovascular bundle, which can add to the complexity of the suture placement [6, 57, 62]. This is further compounded by the relative immobility of the posterior horn compared to its anterior counterpart [39], making it less accessible for manipulation, and difficult to secure the meniscus whilst placing the suture construct. Such challenges can be made easier by the placement of the transtibial centralisation tunnel and stitch midway between the posterior horn and posterior border of the MCL. In fact, except for extrusion at 90°, positioning the centralisation tunnel in this location led to similar results to that of the TTC‐PH technique, with significant improvements in meniscal extrusion (60°) and contact area (90°) compared to the isolated ATPR. However, under load, pathological extrusion was observed at 60° (3.12 mm) and 90° (3.81 mm) at Marker 2. Nevertheless, this does not account for the biological factors such as soft tissue status, muscle contractions, proprioception, healing, and cartilage status in a living tissue. Furthermore, most post‐operative rehabilitation protocols involve 4–6 weeks of light loading prior to full weight bearing [20, 24, 43, 67]. Thus, investigating both biomechanical and biological factors simultaneously will provide a better understanding of the actual effects of the position of the transtibial centralisation on the loading profile of the knee. However, the authors advocate the avoidance of tunnel position behind the MCL due to its vast inferiority in reducing extrusion and improving tibiofemoral contact mechanics across several flexion angles.

### Limitations

This study has several limitations. Although young porcine knees have been used as reasonable surrogates for human knees [8, 42, 55, 56], they are not the same and surgical results may differ. The knee typically has loads applied at numerous angles, not all of which were simulated. However, this study used four angles in keeping with previous studies to represent the typical range to which loads are applied on the knee [2, 23, 28, 49]. Our model used an axial loading scheme, and this scenario is an oversimplification of the conditions in the human knee. However, we expect that more complex loading conditions would only enhance the observed biomechanical effect. We did not use an arthroscopic technique to perform our repairs, and this may have overestimated the observed effect seen with the TTC‐PH construct as the technical challenges described would have been avoided. Whilst previous authors have performed extensive arthrotomies and osteotomies to gain access to the knee, we kept this to a minimum [31, 51]. Although this would have preserved some innate stability, the MCL required resection for insertion of the pressure film, and this may have affected the rotational stability of the knee. However, the effect would be to decrease the contact area and increase contact pressure and extrusion, tending to minimise the potential differences between the intact state, torn state, and various centralisation tunnel repaired states. Fujifilm Prescale film (Tokyo, Japan) was used to measure tibiofemoral contact mechanics. This only provides a measure of tibiofemoral contact mechanics at one time under one set of circumstances, whilst the more commonly used Tekscan pressure sensor (Tekscan, Boston, MA) allows continuous data collection through several load configurations [16, 36, 48].

## CONCLUSION

Combining ATPR with TTC‐PH provides the most appropriate biomechanical properties in terms of reducing extrusion and improving contact mechanics following a MMPRT in porcine knees. When there are concerns regarding its technical applicability, performing the centralisation technique midway between the posterior horn and posterior border of the MCL is a viable alternative.

## AUTHOR CONTRIBUTIONS

*Conceptualization*: Khalis Boksh and Tarek Boutefnouchet. *Methodology*: Khalis Boksh, Randeep Aujla, Michael Hantes, and Tarek Boutefnouchet. *Formal analysis and investigation*: Khalis Boksh, Duncan Shepherd, and Daniel Espino. *Writing, original draft*: Khalis Boksh. *Writing–review and editing*: Duncan Shepherd, Daniel Espino, Arijit Ghosh, Randeep Aujla, and Tarek Boutefnouchet. *Supervision*: Duncan Shepherd, Daniel Espino, Michael Hantes, and Tarek Boutefnouchet.

## CONFLICT OF INTEREST STATEMENT

The authors declare no conflicts of interest.

## ETHICS STATEMENT

Not required. All porcine knee joints material generated for food production.

## Data Availability

The data are available from the corresponding author on request.
